# Spindle Cell Hemangioma and Atypically Localized Juxtaglomerular Cell Tumor in a Patient with Hereditary BRIP1 Mutation: A Case Report

**DOI:** 10.3390/genes12020220

**Published:** 2021-02-03

**Authors:** Jan Papez, Jiri Starha, Pavel Zerhau, Denisa Pavlovska, Marta Jezova, Tomas Jurencak, Katerina Slaba, Martin Sterba, Arpad Kerekes, Tomas Merta, Terezia Haluskova, Hana Palova, Ondrej Slaby, Jaroslav Sterba, Petr Jabandziev

**Affiliations:** 1 Department of Pediatrics, University Hospital Brno, 613 00 Brno, Czech Republic; papez.jan@fnbrno.cz (J.P.); starha.jiri@fnbrno.cz (J.S.); jurencak.tomas@fnbrno.cz (T.J.); slaba.katerina@fnbrno.cz (K.S.); sterba.martin@fnbrno.cz (M.S.); 2 Faculty of Medicine, Masaryk University, Brno, 625 00 Brno, Czech Republic; zerhau.pavel@fnbrno.cz (P.Z.); pavlovska.denisa@fnbrno.cz (D.P.); jezova.marta@fnbrno.cz (M.J.); kerekes.arpad@fnbrno.cz (A.K.); merta.tomas@fnbrno.cz (T.M.); haluskova.terezia@fnbrno.cz (T.H.); sterba.jaroslav@fnbrno.cz (J.S.); 3 Department of Pediatric Surgery, Orthopedics and Traumatology, University Hospital Brno, 613 00 Brno, Czech Republic; 4 Department of Pediatric Radiology, University Hospital Brno, 613 00 Brno, Czech Republic; 5 Department of Pathology, University Hospital Brno, 613 00 Brno, Czech Republic; 6 Department of Pediatric Oncology, University Hospital Brno, 613 00 Brno, Czech Republic; 7 Central European Institute of Technology, 625 00 Brno, Czech Republic; hana.palova@ceitec.muni.cz (H.P.); ondrej.slaby@ceitec.muni.cz (O.S.); 8 Department of Biology, Faculty of Medicine, Masaryk University, 602 00 Brno, Czech Republic

**Keywords:** juxtaglomerular cell tumor, reninoma, spindle cell hemangioendothelioma, kidney, hypertension, children

## Abstract

Spindle cell hemangioma is a benign vascular tumor typically occurring in the dermis or subcutis of distal extremities as red–brown lesions that can grow in both size and number over time. They can be very painful and potentially disabling. A family history of cancer or previous history may be relevant and must be taken into consideration. Juxtaglomerular cell tumor (reninoma) is an extremely rare cause of secondary hypertension diagnosed mostly among adolescents and young adults. Excessive renin secretion results in secondary hyperaldosteronism. Subsequent hypokalemia and metabolic alkalosis, together with high blood pressure, are clues for clinical diagnosis. Histological examination of the excised tumor leads to a definitive diagnosis. Reninoma is found in subcapsular localization, in most cases as a solitary mass, in imaging studies of kidneys. Exceptionally, it can be located in another part of a kidney. Both spindle cell hemangioma and reninoma are extremely rare tumors in children and adolescents. Herein, the authors present a case report of a patient with hereditary BRCA1 interacting protein C-terminal helicase 1 (BRIP1) mutation, spindle cell hemangioma, and secondary hypertension caused by atypically localized reninoma.

## 1. Introduction

Spindle cell hemangioma (SCH) is a benign blood vessel tumor mostly found in the dermis or subcutis of distal extremities. These typically appear as red–brown lesions that, over years, can develop from a single nodule into multifocal painful lesions. There is still little information about the effective treatment of SCH. It is also not yet fully understood why, in some patients, the course of the disease is very progressive [1,2]. Genetic testing of both tumor cells and germline cells can be very helpful in such cases. 

Secondary hypertension (SH) is a more common condition in children compared to adults, who predominantly suffer from primary hypertension. SH is caused by a specific disease entity or another factor, including a wide range of renal diseases, pulmonary diseases, and medications [3]. Accurate identification of SH is very important because many causes are reversible. Hyperactivity of the renin–angiotensin–aldosterone system caused by juxtaglomerular cell tumor (JGCT; also known as reninoma) is one of the possible causes of SH [4]. Co-occurrence of these two rare clinical entities (SCH and JGCT) is extremely rare.

## 2. Case Presentation

**Figure 1:**
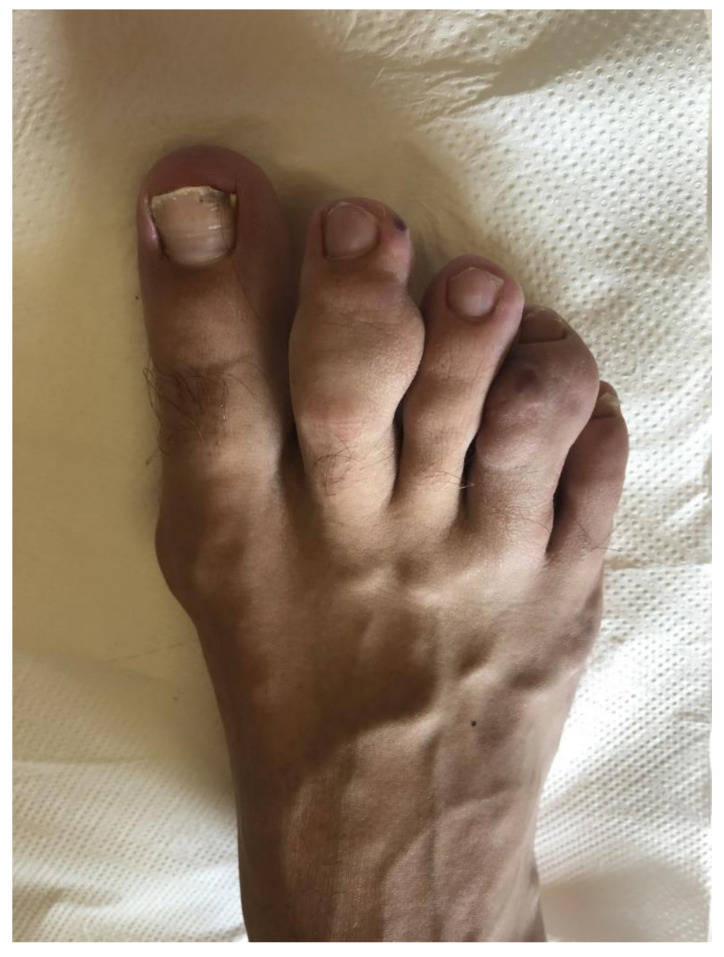
Masses of the spindle cell hemangioma on the toes of the right foot.

We describe here the case of a patient with hereditary BRCA1 interacting protein C-terminal helicase 1 (BRIP1) mutation, spindle cell hemangioma, and secondary hypertension caused by atypically localized reninoma. The boy was born after an uncomplicated pregnancy to nonconsanguineous parents as a full-term twin. His growth and psychomotor development were appropriate. At six years of age, he noticed small painful nodules on his right leg for the first time. Due to the increasing number and mass of the nodules, surgery was performed. Histological investigation of the extirpated tissue revealed spindle cell hemangioma (Figure 1).

Due to the progressive course of the disease, which resulted in numerous plastic surgeries, the boy went through several lines of systemic treatments, including low-dose chemotherapy (vinblastine and methotrexate), sunitinib, and propranolol through the following years. All of the aforementioned treatments were only partially effective in slowing the tumor growth during his pubertal development. Upon reaching adulthood, however, the remaining nodules stabilized in both size and number.

During treatment, the patient’s mother died of gastric carcinoma at 44 years of age. To further confirm the genetic cause of the disease, whole-exome sequencing was performed. A library for whole-exome capture and sequencing was prepared using the TruSeq Exome Kit. The prepared library was loaded onto the NextSeq 500/550 Mid Output Kit v2.5 (150 cycles) and sequenced on the NextSeq 500 instrument (all Illumina, San Diego, CA, USA). The germline exome was analyzed, with findings of pathogenic class 5 mutation c.139C>G/p.P47A in the BRIP1 (BRCA1 interacting protein C-terminal helicase 1) gene in both son and mother. The other family history is unremarkable. Somatic exome analysis of the spindle cell hemangioma showed mutation c.516G>T p.R172S in the IDH2 (isocitrate dehydrogenase 2) gene, which is one of the typical molecular aberrations of spindle cell hemangioma.

**Figure 2:**
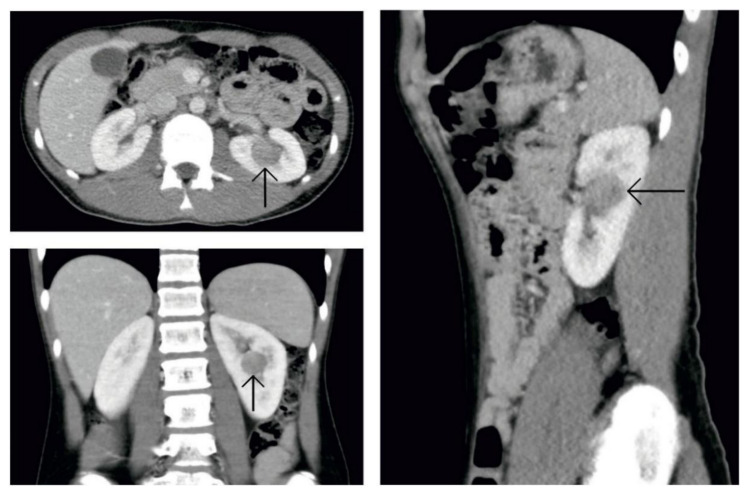
Computed tomography of the abdomen. Picture after administration of an intravenous contrast agent. In a central location of the left kidney is a hypodense round solid tumor.

At 16 years of age, the boy presented no subjective complaints during his regular follow-up care for SCH. Physical examination revealed no pathology except for high blood pressure (190/110 mmHg). He had none of the complaints usually associated with hypertension (e.g., headaches and epistaxis). Echocardiographic examination excluded any possible cardiac cause of hypertension. Nevertheless, mild mitral and tricuspid insufficiency was found as a sign of long-lasting elevated blood pressure. The eye background showed no abnormities. Blood analysis revealed metabolic alkalosis (pH 7.47, bicarbonate 30.9 mmol/L, and base excess +6.1 mmol/L) and asymptomatic hypokalemia (2.9 mmol/L). These laboratory findings, together with hypertension, made us think of hyperaldosteronism. Urinalysis revealed elevated albuminuria (albumin-to-creatinine ratio 46.9 mg/mmol). Ultrasound examination of the abdomen showed a round solid mass in the middle of the left kidney. The patient’s hypertension was managed with a combination of two antihypertensive drugs (calcium channel blockers and α-adrenergic antagonist). With this medication, the patient’s blood pressure dropped gradually below 130/90 mmHg. Abdominal computed tomographic angiography (CTA) excluded renal artery stenosis and confirmed the solid round mass of the left kidney located centrally (Figure 2). Bilateral renal vein sampling for the estimation of plasma renin (PR) level could not be done. PR and plasma aldosterone (PA) levels were measured from peripheral blood. PR was more than 350 ng/L from two different measurements (reference range: 2.7–16.5 ng/L) and PA was 0.832 and 1.001 nmol/L, respectively, also from two different examinations (reference range: 0.12–0.58 nmol/L). These findings proved secondary hyperaldosteronism. 

**Figure 3:**
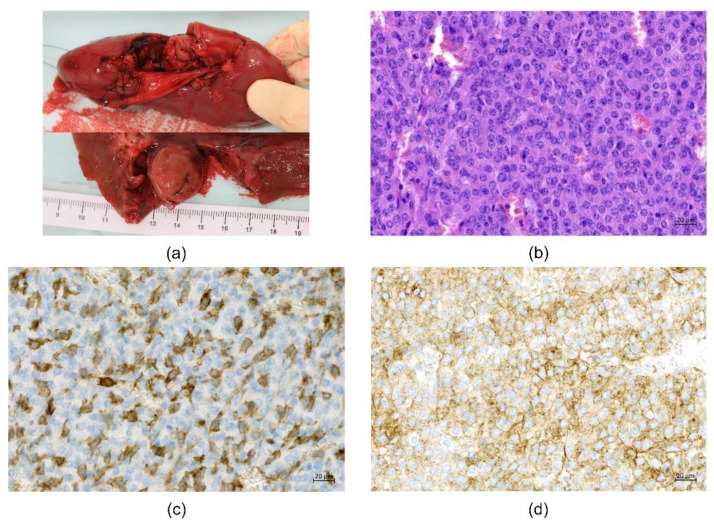
Examination of the tumor. (**a**) Gross examination. Spherical solid pinkish tumor located at the renal hilum (size 25 × 20 × 20 mm). (**b**) Microscopic examination. Monotonous population of polygonal cells with a fine granular cytoplasm and round nuclei in solid sheets and trabeculae. Hematoxylin–eosin staining, magnification 200×. (**c**) Immunohistochemical examination. Positive immunostaining for CD 117, magnification 200×. (**d**) Immunohistochemical examination. Positive immunostaining for CD34, magnification 200×.

The patient then underwent tumor removal with total nephrectomy, which was uneventful. Gross examination showed a sharply circumscribed, spherical, solid pinkish tumor located at the renal hilum (Figure 3a). The tumor was composed of a monotonous population of polygonal cells with a fine granular cytoplasm and round nuclei arranged in solid sheets and trabeculae (Figure 3b). The stroma was richly vascular. There was low mitotic activity (3–5 mitoses per 10 high power fields) and sparse small foci of necrosis without vascular invasion. The definitive pathological diagnosis of benign JGCT was confirmed with positive immunohistochemistry for CD117 (Figure 3c) and CD34 (Figure 3d), as well as smooth muscle actin. Somatic exome analysis of the reninoma revealed no driver mutations or other significant findings. Postoperatively, antihypertensive drugs could be tapered and were withdrawn completely within a few days. Metabolic alkalosis and elevated albuminuria resolved, and serum potassium returned to normal levels. 

After one year of follow-up, the patient remained normotensive and kidney function tests were within normal values. The patient reported no progression of hemangioma lesions and felt generally well.

## 3. Discussion

Spindle cell hemangioma, formerly known as spindle cell hemangioendothelioma, is a rare benign vascular tumor that typically occurs in the dermis or subcutis of distal extremities in children and adults [5]. Tumors appear as red–brown lesions that begin as a single nodule and can develop into multifocal painful lesions over years. Histologically, this tumor is characterized by cavernous blood vessels separated by spindle cells. A significant percentage of spindle cell hemangiomas are completely intravascular with abnormalities in the vein containing the tumor, as well as in the blood vessels apart from the tumor mass. Individuals of different ages can be affected, with no gender predilection [6]. The lesions can be seen in Maffucci syndrome (cutaneous spindle cell hemangiomas occurring with cartilaginous tumors and enchondromas) and Klippel–Trénaunay syndrome (capillary/lymphatic/venous malformations), generalized lymphatic anomalies, lymphedema, and organized thrombus [6]. In Maffucci syndrome, spindle cell hemangiomas are associated with IDH1 or IDH2 mutations [2].

The treatment options that have been used for SCH include surgery, systemic steroids, cryotherapy, laser therapy, radiation therapy, cytotoxic drugs, selective embolization, and recombinant interleukin-2 with varying degrees of success. Because SCH is a benign lesion, the most widely accepted treatment modality is conservative excision without adjuvant chemotherapy or radiotherapy. Following surgical excision, a local recurrence rate of up to 58% has been reported, more commonly in patients with multiple lesions at presentation and near surgical sites rather than within them [5]. This was also the case of our patient.

As mentioned above, spindle cell hemangioma is a very rare benign tumor in children. Considering the family history of gastric carcinoma in his mother at age 44, next-generation sequencing of both the germline and somatic exomes was performed. Germline exome analysis revealed variant c.139C>G/p.P47A in the BRIP1 gene (NM_032043), classified as class 5-pathogenic according to the ACMG/AMG system [7]. BRIP1, or BRCA1 interacting protein C-terminal helicase 1, is involved in DNA repair, which aids the tumor suppressor function of the BRCA1 protein. It is known to be a mutation causing breast cancer [8,9]. This variant has also been described in association with an increased risk of glioma [10] and as a mutation causing colorectal adenocarcinoma, odontogenic carcinoma, ovarian cancer, and hematologic malignancies [11,12]. One may ask if this could explain the limited effect of the treatment during the patient’s growth spurt, the early onset of gastric cancer in his mother, as well as the diagnosis of another rare type of benign tumor in this pediatric patient. 

Somatic exome analysis of the spindle cell hemangioma showed mutation c.516G>T p.R172S (NM_002168, exon 4) in the IDH2 gene, which is one of the typical aberrations found in spindle cell hemangioma. This IDH2 variant has been described in the JAX Clinical Knowledgebase (CKB) as a “gain of function” variant localized within the active site of the IDH2 enzyme [13]. There were no other driver variants found in addition to IDH2.

JGCT is a very rare cause of secondary hypertension. This renin-secreting tumor was first reported by Robertson in 1967 [14]. Since then, approximately 100 cases of JGCT have been reported to date in the English-language literature [15]. The tumors originate from modified smooth muscle cells of the afferent arteriole of the juxtaglomerular apparatus [16]. JGCT affects predominantly women, with a female-to-male ratio of 1.8:1 [17]. The tumor is mostly diagnosed in the second or third decade of life [18], with the age of diagnosis ranging from 6 to 72 years [19]. Reninomas generally have a benign character, and there have been just three reported cases with malignant features. Duan et al. reported the case of a 52-year-old man with metastatic JCGT [20]. Another case report concerned multicentric disease involving the kidneys, spleen, and liver in a 50-year-old man [21]. Shera et al. published the case of an eight-year-old boy with recurrent JGCT [22]. Depending on blood pressure and serum potassium, JGCT can be classified into three types: typical, atypical, and nonfunctioning [23]. The typical type shows high renin with hypertension, hypokalemia, and hyperaldosteronism. The atypical cases present hypertension with normal potassium levels, while the nonfunctioning type demonstrates normal blood pressure and normal potassium levels. Our case was classified as typical. In order to avoid medication targeting the renin–angiotensin–aldosterone system, we used a combination of calcium channel blockers and α-adrenergic antagonist to manage hypertension. This approach allowed us to sample PR and PA without any interference and to disclose secondary hyperaldosteronism from two independent measurements. Moreover, we achieved good blood pressure control using those drugs. Due to hypertension, patients with JGCT usually present with headaches, malaise, epistaxis, and, in younger children, failure to thrive. Other common symptoms include polyuria, polydipsia, nocturia, and myalgia. Our patient presented no subjective complaints, and hypertension was diagnosed before any of the aforementioned symptoms appeared. Unlike our patient, some reported cases present longer histories of headaches with delay in diagnosis [15,17,24]. Imaging studies of the uropoetic system should be performed as soon as hypertension is detected in children. In the case of JGCT, ultrasonography of the kidneys usually shows a hypoechogenic mass [25], but small tumors (less than 10 mm) can easily be overlooked [26]. CTA is generally considered the best technique for imaging evaluation of reninomas, with a reported 100% sensitivity in diagnosing even small tumors [24]. Renin sampling from both renal veins (lateralization) can help to localize the site of renin production [16,27]. It is necessary to perform this examination in case the lesion is not clearly visible on imaging studies. We did not carry this out because the tumor was identified precisely by CTA. Moreover, there are reports of renal vein catheterization failing to provide adequate localization of a superficial tumor due to it being drained by peripheral veins and not by a renal vein [28,29]. A major curative modality in the case of reninoma is surgical resection of the tumor, thereby resolving hypertension in most patients. There are sporadic cases reported in the literature of persistent hypertension after resection of the tumor [28,30]. The etiology of hypertension in these reports was thought to be due to cardiological changes from longstanding hypertension or other causes of hypertension.

Nephron-sparing surgery is today considered the standard treatment option because of the usually small size and subcapsular location of reninomas [25,31,32]. It is recommended to remove an adequate rim of normal kidney tissue (at least 0.5 cm) [32]. In the case described here, the tumor was found during surgery in an atypical central location of the left kidney (as described by imaging studies). Although we wanted and tried to remove the reninoma by nephron-sparing surgery, because of its proximity to renal vessels, very strong surrounding perfusion, and unclear border of the tumor that could cause life-threatening bleeding, we decided to perform a total nephrectomy.

JGCT is not the only renin-secreting tumor [33]. This hormone is also produced by renal cell carcinoma, hemangiopericytoma, or Wilms’ tumor. Histopathological examination is therefore necessary for definitive diagnosis of the reninoma. Grossly, JGCTs are usually well-circumscribed, small tumors with a complete or partial fibrous capsule, with a yellow-to-gray–tan surface [33]. Histologically, the tumor is composed of closely packed, uniform round-to-polygonal cells with a granular, eosinophilic cytoplasm, and round-to-oval nuclei with few mitoses [34]. Neoplastic cells are positive for CD34, CD117, smooth muscle actin, and vimentin and negative for cytokeratin, desmin, protein S-100, HMB-45, chromogranin, and synaptophysin [33]. A few reports have been directed to genetic abnormalities in JGCT. In the study of Kuroda et al., gains were shown of chromosomes 3, 4, 10, 13, 17, and 18, as well as a loss of chromosome 9 [35]. Two cases showed an aneuploid karyotype and complex genomic imbalance [36,37].

## 4. Conclusions

Spindle cell hemangioma and reninoma are both rare types of tumors. The odds of finding these two benign tumors in a single patient are extremely low. Therefore, germline exome analysis was performed and a mutated variant of the BRIP1 gene was found. This variant is generally linked to tumor growth and its rapid progression. This may have some relation to the quite progressive course of SCH and the rather unusual localization of reninoma in our patient. Reninoma is typically found in the subcapsular region. In most imaging studies of kidneys, it is seen as a solitary mass. Long-term observation and regular checkups in this patient with SCH helped us reveal severe hypertension as a result of reninoma before any other clinical symptoms presented.

This case draws attention to the importance of treating a patient holistically and regularly reassessing family history in such chronic patients. Connecting initially unrelated diseases using exome sequencing may provide us more information and help us in coming up with a new treatment strategy.

## Data Availability

The data presented in this study are available on request from the corresponding author. The data are not publicly available due to privacy and ethical restrictions.
